# Cost-effectiveness of malaria elimination in Sampov Loun Operational District, Cambodia

**Published:** 2020-04-01

**Authors:** Ir Por, Siv Sovannaroth, Alexander Moran, Lek Dysoley, Sokomar Nguon, Om Bunthy, May Sak Meas, Lawrence Barat, Rida Slot, Sharon Thangadurai, Bryan K. Kapella, Saad El-Din Hassan, Ly Po, Sen Sam An, John E. Gimnig, Mary McDowell, Michael Thigpen, Jennifer Armistead, Hala Jassim AlMossawi, Soy Ty Kheang, Neeraj Kak

**Affiliations:** 1 National Institute of Public Health, Phnom Penh, Cambodia;; 2 National Malaria Control Program, Phnom Penh, Cambodia;; 3 University Research Co., LLC (URC), Chevy Chase, MD, USA;; 4 PMI/USAID Cambodia Malaria Elimination Project, Phnom Penh, Cambodia;; 5 Sampov Loun Operational District, Battambang Province, Cambodia;; 6 US President’s Malaria Initiative, United States Agency for International Development,Washington DC, USA.

## Abstract

**Background:**

Over the past decade, Cambodia has seen a significant decline in its malaria burden. The government has established the goal of eliminating malaria in the country by 2025. With PMI/USAID support, Cambodia is implementing a package of interventions as part of its efforts. This assessment aimed to describe the cost of malaria elimination activities in Sampov Loun Operational District (OD) between July 2015 and March 2018, to describe the cost per malaria case detected under PMI programming, and to estimate the incremental cost-effectiveness of the elimination programme per *Plasmodium falciparum* (*Pf*) or *P. vivax* (*Pv*)/*Pf* mixed case averted under the Cambodia Malaria Elimination Programme (CMEP) and the U.S. President’s Malaria Initiative. Opportunity costs of government workers were also assessed to understand the theoretical cost of sustaining this programme through government efforts alone.

**Materials and methods:**

We conducted an empirical micro-costing analysis based on elimination activities alone using CMEP internal project implementation data and corresponding epidemiologic data from July 2015 to March 2018 and empirical findings from implementation to date. We then constructed a cost model in Microsoft Excel using empirical data and used a cost-effectiveness decision tree to describe programme effectiveness in the first three years of implementation and to estimate efficacy for the subsequent year.

**Results:**

The total cost of malaria elimination activities in Sampov Loun OD from July 2015 to March 2018 was $883,096. The cost per case of malaria detected in 2017 was $1,304. Including opportunity costs for government staff from July 2015 to March 2018, the total cost was $926,000. Under continued CMEP implementation, the projected future total cost of the program would be about $110,000 per year, or $0.64 per Sampov Loun resident. The incremental cost-effectiveness of the elimination programme was $28 for every additional *Pf* or *Pv/Pf* mix malaria case averted, compared to the no-CMEP proxy.

**Conclusion:**

CMEP activities are cost effective compared to the no-CMEP proxy, as shown through an incremental cost-effectiveness of $28 for every additional *Pf* or *Pv/Pf* mix malaria case averted. The total cost of the project is 0.93% of the total per capita spending on health in Cambodia and about 5% of all government health expenditure. Continuing investments in malaria will be needed at national level for stewardship and governance and at local level for ensuring programme readiness in case of malaria outbreaks.

## Introduction

The US President’s Malaria Initiative (PMI) Control and Prevention of Malaria (CAP-Malaria; 2011-2016) and CMEP (2016-2021) support the Cambodian National Center for Malaria Control, Parasitology and Entomology’s (CNM) goal of nationwide malaria elimination by 2025. PMI-funded malaria elimination activities, including CAP-Malaria and CMEP, seek to develop a scalable, evidence-based elimination model in Sampov Loun (SPL) operational district (OD) and to support its dissemination and replication for malaria elimination in Cambodia. PMI-funded malaria elimination activities began in July 2015 in Sampov Loun Operational District (SPL OD), Battambang Province.

In Cambodia, there has been a decline in confirmed malaria cases from 8/1,000 people in 2006 to 1/1,000 people in 2016 [1]. This decrease can be attributed to widespread use of effective artemisinin-based combination therapies (ACT), increased access to long-lasting insecticide-treated nets (LLINs), and early diagnosis and appropriate treatment (EDAT). Additionally, behaviour change communication (BCC) services, particularly through the network of village malaria workers (VMWs), established in 2004 under the auspices of CNM [2], and huge effort made by public health facilities (HFs), operational districts (ODs) and provincial health departments (PHDs) have contributed to this decrease. Building on the Malaria Elimination Action Framework 2016-20 (MEAF), approved by CNM on 20 May 2015 [3], Cambodia committed to the goal of reducing malaria incidence to less than one per 1,000 people at risk in each OD in the country and eliminating *Pf*, including multidrug resistance, by 2020.

Since March 2016, no locally transmitted cases of malaria have been detected in Sampov Loun OD. The majority are among SPL residents who travel outside of SPL for short-term work in the forestry or agricultural sector with some cases among migrant workers coming into SPL OD for temporary employment. Currently, most cases reported are among young males (15-49 years of age) who live or work in forested areas or on private farms near forests. With PMI support, from 2015-2016, the number of malaria cases in supported areas decreased by 57 percent. In recent years, outbreaks have occurred in geographic areas with high migration as people move due to land clearance for agricultural settling or other commercial purposes. In addition, increasing artemisinin resistance, detected following the failure of artesunate-mefloquine combination therapy (ASMQ) in 2008, and then rapid development of molecular signs of resistance to Malarone (atovaquone-proguanil) in 2013 [4], and rapid development of treatment failures to dihydroartemisinin-piperaquine (DHA-PIP) reported in 2016/17, have made the goal of achieving malaria elimination in Cambodia challenging.

Since 2017, CMEP has worked closely with stakeholders to continue implementation of malaria elimination activities in SPL OD, which were begun under the predecessor project CAP-M, and has supported introduction of malaria elimination activities in four additional ODs: Battambang, Maung Russey, Thmar Kaul and Pailin. This study is an activity-based empirical micro-costing analysis using data reported by the CMEP Project team. We examine the cost of USAID/PMI-funded malaria elimination activities in Sampov Loun OD, assess government opportunity costs of implementing the program, and project the incremental cost effectiveness of additional *Pf* and *Pv/Pf* mix malaria cases averted under CMEP implementation compared to a no-CMEP proxy.

## Materials and Methods

PMI-funded malaria elimination activities began in SPL OD in July 2015. The start-up period included July-December 2015 with 80% coverage, and full implementation (100% coverage) began in January 2016. PMI-funded malaria elimination activities use an OD-centered approach to build capacity for ongoing malaria control and elimination. The project team supported development, implementation, and monitoring of OD annual operational plans, introducing data visualisation to support evidence-based decisions. Joint supervision at all levels were used to improve the quality of care and routine reporting. To increase inclusion of at-risk-populations, the project team worked with community networks for better access to and quality of early diagnosis and treatment (EDAT). It also strengthened passive and active surveillance systems, supported distribution of LLINs, and implemented behaviour change communication (BCC) activities to promote prevention and treatment-seeking behaviours.

A specific example of these activities is the 1-3-7 approach to elimination. Briefly, this approach aims to identify suspected malaria cases by VMWs or at HFs. All suspected cases are tested via either RDTs or microscopy on day one. Patients with negative results are advised to seek consultation at a public health facility, and patients with positive results are immediately placed on a three-day directly observed therapy (DOT) regimen with ASMQ or similarly appropriate therapy for pregnant women or people with drug resistance. All confirmed malaria cases are reported to OD health authorities within one day of diagnosis. Within three days, case investigations, which include interviews with index cases and resultant case classification, are conducted by village malaria workers and health facility staff. Finally, within seven days, targeted response measures are undertaken, although these often happen within three days in conjunction with case investigation activities. These response measures included reactive case detection (initially testing of co-travellers, household contacts and family members, and surrounding households with suspected malaria cases, and later only testing of co-travellers and family members after no additional cases were identified in other groups), and provision of health education and insecticide-treated nets. Additionally, 28-day follow up microscopy is conducted for all confirmed *Pf* and *Pf/Pv* mix cases to assess treatment efficacy.

### Cost of malaria elimination activities in Sampov Loun OD

The list of activities included for determining costs is limited to elimination activities rather than activities that include control and prevention measures. Therefore, the list includes certain activities conducted under the CAP-Malaria Project, which was extended to January 2017. Cost estimates were based on activities focused exclusively on malaria elimination and were defined based on the classifications shown in Table 1. Financial data are reported in inflation-adjusted 2018 $US based on the average inflation rate of 3% prevailing in Cambodia for the period [5].

**Table 1:** Definition and classification of elimination activities.

Cost item	Definition	Examples
Project launch	Costs incurred early to develop and establish the programme. These were not recurrent costs.	Supervision tool training to HCs on VMWs, private sector field assessment, malaria case investigation training.
Management (other than actual programme implementation)	Costs for meetings and other communication with CMEP management and other managers in the health system.	Feedback workshop on technical supervision, Routine Data Quality Assessment (RDQA) and Annual Operational Plan (AOP) review, OD Malaria Supervisor (ODMS) & Operational District Director Attendance at AOP Y 2015 Final.
Field team (OD staff, supervisors, laboratory support)	Costs for ODMSs, VMW, HF and provincial staff supervision and training conducted in the field.	Day 3+ surveillance training, training to Provincial Health Department (PHD) laboratory on blood smear reading & slide collection from HCs to Referral Hospitals (RHs).
Case investigations and management by VMWs/Health Care Workers (HCWs)	Costs of health workers investigating a confirmed malaria case identified in SPL. The difference between this and the above categories is that it includes only costs on a per-case-identified basis.	Intensified case management, investigation and classification (imported/indigenous), including 28 days follow-up for *Pf* and mix cases.
Laboratory	Costs for laboratory support such as reagents, Rapid Diagnostic Tests (RDTs), ACTs and other supplies. It does not include training of laboratory staff.	Purchase of RDTs to bridge gaps by PMI-Global Health Supply Chain – Procurement and Supply Management (GHSC-PSM), needed laboratory supplies to bridge gaps*.
LLINs (BCC, distribution)	Cost of LLIN distribution and any BCC activity or material distributed or available to the public for malaria education.	Billboard instalment at key entry points to the OD (about three large billboards), Cross-border education to migrant population by sound system.
Other indirect project costs	Costs for activities/supplies important for operation of the project not included above.	Office supplies, copying and mailing, etc.

*Routine supply of RDTs and ACTs by CNM was considered malaria control, but additional supplies provided by PMI-funds to bridge gaps was considered malaria elimination.

Project staff costs were estimated based on URC accounting records. The level of effort (LOE) was estimated as a total percentage of full-time equivalents (FTEs) for each role involved and reported in groups according to where the roles were based – central, provincial or operational district. Government worker costs were estimated at two levels. The first level was the estimate of the proportion of FTEs involved in the elimination activities as they occurred from 2015 onward. The second was the estimate of the FTEs required if the government staff were implementing all activities for elimination without input from the CAP-M/CMEP Project Staff. These estimates were considered opportunity costs. The government did not recruit additional personnel for their contribution to elimination activities but used those already working in the malaria programme. Implementation of elimination activities meant reassigning staff from their normal duties to those that were part of elimination activities. For this reason, we assigned values to their contribution even though this did not impact the government’s labour budget. We considered it important to capture the value of their time they would otherwise be spending doing other government work duties.

Treatment costs for malaria cases were based on estimates of averages based on empirical evidence from the project and included management of the proportion of cases developing side effect/adverse events from first line artemisinin-based combination therapy/single low-dose primaquine (SLD PQ) requiring further management.

Epidemiological data was collected from routine monitoring by CMEP. These data include the number of suspected malaria cases tested, the number of confirmed malaria cases and the proportion infected by different parasites – *Pf*, *Pv* or *Pf/Pv* mixed infections (Table 2). The number of cases averted was calculated by determining the difference between the number of cases reported through routine monitoring and the number of cases reported in the year before implementation of the elimination programme began. For the purposes of this analysis, *Pf* cases were combined with *Pf/Pv* mixed cases.

**Table 2:** Malaria cases in Sampov Loun, 2015 to 2018.

	2015*	2016	2017	2018*
Population	161,663	162,472	170,735	173,583
Tests of suspected cases	6,940	4,915	6,738	13,436
Confirmed cases (all forms)	519	206	181	184
Confirmed *Pf/Pv* Mix cases	31	4	3	0
Confirmed *Pf* cases	195	82	43	15
Confirmed *Pv* cases	293	120	135	168

*2015 and 2018 were annualized based on partial year data.

A model was developed in Microsoft Excel, based on the empirical cost data classified on the list of activities conducted for malaria elimination (Table S1). The ‘Project Activities’ part of the model separates the cost into years from 2015 to December 2017 and divides them into seven categories. Examples of capacity development costs include those for training for provincial malaria supervisor OD malaria supervisors (ODMSs), HF staff and VMWs. Case-based costs include those dependent on the number of people confirmed to have malaria, what species of parasite they were infected with and whether their infection was resistant to first line drug therapy. The average case-based cost is based on empirical data collected from the project between 2012 and 2018 with the first three years classified as the pre-elimination period which served as the proxy in the cost-effectiveness analysis for the counterfactual of no elimination activities in operation in Sampov Loun.

**Table S1:** Chronology of programme activities.

Year	2015		2016				2017				2018	
Quarter	3	4	1	2	3	4	1	2	3	4	1	2
Coverage (%)	80		100									
Consultative workshop/planning (before July 2015)												
Pre-implementation situation analysis (CMEP Objective 1, Task 1)						●						
Support AOP	●				●				●			
Private provider mapping		●				●						
Update/revise all elimination related SOP & tools									●		●	
Support Special Working Group for Malaria Elimination (District)	●	●	●	●	●	●	●	●	●	●	●	●
Support Special Working Group for Malaria Elimination (Province)		●		●		●		●		●		●
Support National Malaria Elimination Working Group/Task Force (Meetings were organized at initial stage of CAP-Malaria)												
Case management & EDAT training to health facilities	●								●			
Case management & EDAT training to VMWs	●									●		
Case management & EDAT training to private providers	●									●	●	
Bimonthly supervision visits for PPs	●						●	●	●	●	●	●
Bimonthly meetings with PPs	●		●		●		●	●	●	●	●	●
Monthly meetings with VMWs	●	●	●	●	●	●	●	●	●	●	●	●
Structural supervisions to VMWs (2times/year)			●		●		●		●		●	
Visit to VMWs absent at monthly meeting (5% of all VMWs)		●	●	●	●	●	●	●	●	●	●	●
3-day program management course with PHDs and other ODs									●			
M&E training to HF staff (introducing M&E form)	●							●				
Entomology capacity development for OD malaria supervisors											●	
Tech. agement, supervision HIS/MIS, from stock PHD/status, ODs lab to service) HF (planning, case man-		●	●	●	●	●	●	●	●	●	●	●
Test kits to VMWs	●	●	●	●	●	●	●	●	●	●	●	●
ACT reallocation (transport)	●	●	●	●	●	●	●	●	●	●	●	●
3 days of DOT by VMWs for Pf/Pv and mixed cases	●	●	●	●	●	●	●	●	●	●	●	●
28-day follow up of Pf/Mixed cases (D0 & D28 slide prep/reading)	●	●	●	●	●	●	●	●	●	●	●	●
Single-low-dose primaquine (D7 visits to monitor adverse events)									●	●	●	●
Financial support to hospitalized patients for 2nd line treatment	●	●	●	●	●	●	●	●	●	●	●	●
5-day training on NCA/ICA by CNM lab unit									●			
Quarterly supervisions by provincial lab supervisor to HF		●	●	●	●	●	●	●	●	●	●	●
Support for national surveillance technical working group	●	●	●	●	●	●	●	●	●	●	●	●
3-day Training on surveillance for malaria elimination	●				●				●		●	
Regular case notification through SMS with mobile apps/devices	●	●	●	●	●	●	●	●	●	●	●	●
Training to use mobile apps/devices											●	
Case investigation (HF staff & VMWs to visit patient homes)	●	●	●	●	●	●	●	●	●	●	●	●
Reactive members case detection: RDT for index case, co-travelers & HH	●	●	●	●	●	●	●	●	●	●	●	●
IRS (happened only at initial stage with CNM supplies)												
ITN distribution in 3-year cycle	●										●	
ITN continuous distribution via buffer and topping up approach (3 days/month, VMWs visit households/farms)	●	●	●	●	●	●	●	●	●	●	●	●

The cost-effectiveness model was used to estimate the efficiency of the programme in the first three years of operation of the elimination programme, then estimated for a subsequent year based on 2018 results from January 2018 to March 2018. This estimate was constructed through assuming one scenario with project staff involvement and one without (i.e., government staff implementing all elimination activities). The estimate included the cost of maintaining active case detection and investigation but did not include foci investigation.

The cost model was constructed in Microsoft Excel with the empirical data provided for the descriptive analysis of the costs incurred by the programme. Values in the model were then changed to determine the relative effect of changes in inputs on the overall programme cost for deterministic sensitivity analysis, based on what percent change in one input at a time would have on the percent change in the main outcome.

## Results

The total cost of malaria elimination activities in Sampov Loun OD from 2015 to 2017 was $883,096 (Table 3). This included all elimination-specific activities (Table S1) over this period from both PMI-and Global Fund/ United Nations Office for Project Services (GF/UNOPS)-funded malaria elimination programming. The estimated cost to maintain malaria elimination status beyond the period of observation is approximately $236,000 per annum, or less than $1.36 per resident in the OD if the programme was to be continued with the current implementing partner.

**Table 3:** Total PMI and GF/UNOPS-funded elimination costs (in USD) and Level of Effort (LOE) by year and category, 2015-2017.

Categories	2015		2016		2017		Total	
	Cost (start-up)	LOE	Cost (full implementation)	LOE	Cost (continuation)	LOE	Cost	LOE
CMEP Start-up	22,689	7%	0	0%	0	0%	22,689	3%
Management (excluding program implementation)	2,086	1%	226	0%	11,046	5%	13,358	2%
Field team (OD staff, supervisors, lab support)	106,448	31%	103,366	34%	106,994	45%	316,807	36%
Investigations (VMW/HC allowances)	35,024	10%	46,608	15%	8,973	4%	90,605	10%
Laboratory supplies	2,750	1%	2,750	1%	2,750	1%	8,25	1%
LLINs (distribution, BCC for use)	96,467	28%	78,083	25%	25,980	11%	200,529	23%
Indirect	1,995	1%	3,815	1%	4,659	2%	10,469	1%
Project labour estimate	71,237	21%	73,441	24%	75,712	32%	220,391	25%
**Total**	**338,695**		**308,287**		**236,114**		**883,096**	

The total cost of the programme per case detected was $653 in 2015, $1,497 in 2016 and $1,304 in 2017, with an average over the whole period of $975 per case detected. The cost is projected to decrease to $603 per case detected with the same model of implementation or $472 per case detected with the government implementing the programme, assuming the same level of success as the present programme. The cost per case detected will increase as the occurrence of malaria becomes rare.

The labour cost of CMEP staff totalled $75,813 for 2018. Most of these costs were incurred at the OD level. When including opportunity costs of the government staff at the OD, PHD and central level for the three-year period of CMEP to date, the total cost for the programme is $897,000 with current CMEP implementing partner. Beyond this period, assuming continued CMEP implementation, the total cost of the programme, including government staff opportunity costs, would be approximately $110,600 per year or $0,64 per Sampov Loun resident.

The incremental cost-effectiveness of the elimination programme is presented in Table 4, which describes cost-effectiveness results under the three different scenarios (With CMEP and no government costs; With CMEP plus government costs; Government only), in which all results are compared to a no-CMEP proxy. For example, in 2018 the estimated cost of CMEP implementation was $253 for every additional malaria case averted (of any kind), compared to the no-CMEP proxy. Examining *Pf* or *Pf/Pv* mix specifically for 2018, the estimated cost of CMEP implementation was an additional $28 per *Pf* or mixed case identified compared to the no-CMEP proxy. Finally, the estimated cost of CMEP implementation was $310 for each additional *Pv* case averted in 2018 compared to the no-CMEP proxy. These values should be considered with caution, however, in that the individual rows (i.e. *Pf*/mix and *Pv*) should not be summed to arrive at a ‘grand total’ because relative treatment costs, populations and other factors have created these summary values.

**Table 4:** Cost-effectiveness analysis results.

ICER expressed by:	CMEP only				government CMEP plus costs				Government only
Year	2015	2016	2017	2018	2015	2016	2017	2018	2019
$/malaria case averted	215	195	206	253	216	196	207	255	242
$/Pf or mixed case averted	24	22	23	28	24	22	23	29	27
$/Pv case averted	260	238	252	310	261	239	253	312	297
$/hospitalization averted	4,711	4,142	4,348	5,267	4,729	4,154	4,364	5,299	5,040

The deterministic sensitivity analysis for the 2018 model showed that the probability of symptoms had the greatest impact on the cost-effectiveness of the intervention (Table 5). A 1% increase in the probability of a person presenting with malaria symptoms, and therefore requiring an RDT, would give a 0.52% increase in the cost of the programme and therefore a 0.52% decrease in its cost-effectiveness.

**Table 5:** Sensitivity analyses.

Variable	% Change from 1% increase
**Cost**	
Cost of malaria RDT	0.165
Cost of treating *Pf*	-0.032
Cost of treating all malaria	-0.061
Cost of treating *Pv*	-0.029
Cost of CMEP	0.242
**Probability**	
Probability of symptoms requiring testing	0.519
Probability of *Pf* / mixed	0.146
Probability of *Pv*	0.161
Probability of all malaria	0.163
Probability of hospitalization	0.144

## Discussion

The total cost of malaria elimination activities in Sampov Loun OD from July 2015 to March 2018 was $883,096. This includes all activities specifically aimed at eliminating malaria over this period but did not include malaria prevention and control activities from the CAP-Malaria Project. It is estimated that the cost to maintain the malaria elimination status beyond the period of observation is approximately $110,600 per annum total or less than $0,64 per resident in the OD if the programme was continued with the current implementing partner. This is about 0.93% of the total health expenditure expected for the 173,000 people of Sampov Loun OD [6]. The large difference between *Pf* and *Pv* case cost effectiveness was driven by the cost of treatment of *Pf* cases being almost a factor of 10 higher than for cases of *Pv*. That is, applying the same set of interventions to the relatively cheaper-to-treat *Pv* cases means that additional cases of *Pv* identified are less cost-effective than identifying expensive-to-treat *Pf*/mix cases.

When the opportunity costs of the government staff at the OD, PHD and central level are included, for the three-year period of PMI-funded elimination programming, the total cost of the programme is $897,000 with the same CMEP partners leading the implementation. Beyond this period, with CMEP still leading the implementation, the total cost of the programme, including government staff opportunity costs, would be approximately $241,000 per year or $1.39 per Sampov Loun resident, which is still less than 1% of the total health expenditures in Sampov Loun and approximately 5% of total government spending on health. This does not include the cost savings realised with the decrease in expenditures on malaria treatment and lost productivity.

The cost savings realised by cases of malaria averted were not part of this calculation. The cost to the health system for treating *Pf* or *Pv/Pf* mix malaria cases within the programme were about $21.50 for those treated as outpatients (OPD) and $104.50 for those requiring hospitalisation (IPD). If lost productivity of those with malaria was included, the cost per case would be $146 (OPD) and $230 (IPD) [7,8]. Using the historical average of 12% hospitalisation for *Pf* and *Pf/Pv* mix cases, if the programme was responsible for averting about 7,500 of such cases per year, it would be cost-neutral to society in Sampov Loun OD.

This cost-effectiveness analysis was focused exclusively on systems-level inputs, rather than examining cost and efficiency from the societal perspective. The intervention would have appeared more attractive by including costs borne by all parties including the government, donors and Cambodian citizens, especially if the positive externalities of a geographical area being classified as malaria-free over the long term were accounted for.

Sensitivity analysis for the 2018 model showed that the model is most sensitive to changes in the proportion of those being tested for malaria. This is related to the increase in the testing that occurred in 2018 to achieve the recommended annual blood examination rate of 8%. Of note is the finding that a unit (1%) increase in the cost of CMEP implementation only increases the overall costs of the elimination activity by 0.24%.

It is likely that the cost of maintaining the status of being malaria-free in Sampov Loun will decrease over time because the high intensity capacity development training may not be necessary in perpetuity. If the malaria elimination training content was integrated into health provider continuing education activities, there would likely be lower costs as this method of malaria management becomes institutionalised. It is possible that expenditure on LLINs and BCC may decrease as the information and supplies for vector control and malaria control reaches saturation, and as malaria transmission is interrupted and the need for LLINs, except for forest workers, diminishes.

The labour costs with government intervention were based on projections under the assumption that a lower-case burden would occur concurrently with handing over CMEP activities to government staff assuming their capacity to conduct these tasks efficiently. The level of effort (LOE) required by the CNM Director at the central level was estimated at 3%, assuming that while this is a low proportion of their activities, their input is of great importance for accountability and good governance.

We showed the cost of the programme both with and without inclusion of the government workers time involved in implementation of elimination activities. The actual cost to the government of this time was essentially zero because they did not increase the number of staff to conduct elimination activities. Activities were completed with the same workforce as before but with a shift in malaria management duties from one set of activities to elimination-specific activities. However, these costs were included in part of the analysis to give an inclusive calculation of the costs, relevant especially if implementation of the same programme was to occur in another setting where additional government-paid workers would be needed for implementation.

According to programme data, there were 184 imported cases to Sampov Loun, mostly from other locations in Cambodia (some from Thailand), compared to 0 indigenous cases in 2018. If the intervention was conducted in other parts of the country, the number of cases overall in the country would decrease, assuming the programme was implemented successfully. If this was the case, it would likely substantially decrease the number of imported cases and therefore decrease the case-based costs of implementation in this OD.

This was a retrospective examination of the cost of the elimination programme. While total expenditures of the programme were captured accurately, it was difficult in some cases to define an activity as one for control and prevention of malaria or one specifically for elimination. This is especially the case as the CAP-Malaria project activities in 2015 as the programme in Sampov Loun transitioned from control/prevention to elimination.

Several activities included in the costing involved participation of health provider staff and managers from other ODs in the same province. This may have increased the cost of the activity compared to if these capacity development activities had been provided exclusively for Sampov Loun staff. For example, a larger venue and greater time may have been required for meetings for capacity development training with participants from several ODs compared to the cost for the same meeting with the smaller Sampov Loun group. We included the whole cost of such activities as they occurred because this is an empirical costing exercise. Given the training did include staff from other ODs, the marginal cost of implementing the same programme in these ODs within the same province will be lower.

There will likely be variation in the cost of implementing the same programme outside this geographical area. Health providers and managers in Sampov Loun OD and Battambang Province had extensive experience with donor-funded malaria control activities over the last decade. This previous investment may have made this implementation more efficient than it would have been in areas where health workers and managers had less experience with implementation of specific malaria control programmes. Also, differences in population size and density, accessibility and infrastructure, malaria prevalence, and mosquito vector and environmental issues may all change the cost of implementing the programme in other circumstances. However, we expect that the costs reported here would be in the same order of magnitude if implemented outside Sampov Loun.

This cost analysis did not include externalities of the programme including the benefits to improve overall health system functioning. Decreasing the burden of malaria cases on health facilities allowed health facility staff to dedicate more time to clinical areas other than malaria. It is also possible that the malaria elimination programme may have had a positive effect on the incidence and response to dengue as a consequence of better vector control and surveillance of those with fever [9-11]. We also did not include externalities of reducing the risk of spread of artemisinin-resistant malaria, which also has important public health implications nationally and internationally [12, 13].

Entomological foci investigation is recommended by the World Health Organization for malaria elimination in Cambodia [14, 15]. It involves comprehensive activities including mosquito capture and microscopic analysis at a central laboratory. This analysis did not include this component as part of the elimination strategy because it was not part of CMEP implementation, and no residual foci were identified in Sampov Loun.

An important factor in the reduction of the transmission of malaria is the environmental changes occurring in the region that affects Sampov Loun substantively. Chief among these is deforestation, which adversely affects mosquito vector populations and over the long term, reduces the number of workers labouring in the forests, thereby exposing themselves to malaria infection. Changes in rainfall patterns may also reduce the vector population, contributing to a reduction of malaria transmission. Clearing of forested areas in and around Sampov Loun may have contributed to the decline in mosquitoes and a diminution of the risk of malaria prior to the implementation of elimination activities. While it was beyond the scope of this analysis to consider the economics of these changes, they are factors that can affect the cost and efficiency of the malaria elimination programme.

## Conclusions

The programmatic cost of malaria elimination as implemented under the USAID CMEP Project and partly in the predecessor CAP-Malaria Project in Sampov Loun was $883,000 between 2015 and 2017. The annual projected cost for 2018 and beyond is approximately $110,600 or $0.64 per capita. These figures do not account for the additional economic benefits realised from the burden of malaria averted or the positive externalities of the programme. The cost of the project is 0.93% of the total per capita spending on health in Cambodia, reported to be $69 per year in 2014 [7]. Considering only government health spending reported in 2014 of $12.77 per person per year, the programme cost for 2018 and beyond would be 5% of that spending for the 173,600 people in Sampov Loun [7]. Based on these results, we conclude that CMEP elimination activities are cost-efficient and confer additional effectiveness compared to the standard government implementation of malaria control based on estimated budgets in the MEAF 2016-2020. Moving forward, these innovative CMEP elimination strategies should be further integrated into ongoing government practice to institutionalise elimination activities and to realise a malaria-free Cambodia.
